# The Influence of Radioligand Therapy on Immunogenicity Against SARS-CoV-2—A Retrospective Single-Arm Cohort Study of Metastatic Prostate Cancer Patients Receiving PSMA Radioligand Therapy †

**DOI:** 10.3390/cancers17111865

**Published:** 2025-06-02

**Authors:** Carsten S. Kramer, Aleksandr Eismant, Aditi Mishra, Corinna Müller, Christian Landvogt, Richard P. Baum

**Affiliations:** CURANOSTICUM MVZ GmbH Wiesbaden–Frankfurt, Center for Advanced Radiomolecular Precision Oncology, Aukammallee 33, 65191 Wiesbaden, Germany; carsten.s.kramer@gmail.com (C.S.K.);

**Keywords:** SARS-CoV-2, vaccination, COVID-19, PSMA, radioligand therapy, clinical care, prostate cancer, immunization, humoral response

## Abstract

**Background**: Severe acute respiratory syndrome coronavirus 2 (SARS-CoV-2) has become a rising threat for immunocompromised cancer patients. The reduced immune defense may be a result of the malignancy itself or a side effect of therapy. While many chemotherapies can severely diminish the effect of vaccines against SARS-CoV-2, the effect of radioligand therapy has not yet been studied so far. **Methods**: In our database, 64 patient records of patients with metastatic castration-resistant prostate cancer that were treated with PSMA-directed radioligand therapy (PRLT) were randomly selected and checked for specific information (vaccination status, past corona virus disease 2019 (COVID-19) infections, the period between PRLT and vaccination, and antibody titers). A total of 30 patient records had sufficient information to examine the interference between PRLT and the vaccination against SARS-CoV-2. **Results**: In the analyzed cohort, 96.7% of the patients achieved seroconversion after receiving—on average—the third (booster) vaccination against SARS-CoV-2 and two PRLT cycles with average administered activities of 16.1 ± 7.2 GBq (435.1 ± 194.6 mCi) of lutetium-177 and 13.7 ± 6.6 MBq (0.37 ± 0.18 mCi) of actinium-225 (as part of ‘TANDEM therapies’) per patient. **Conclusions**: In the reviewed population, neither the initial response nor the maintenance of a positive immune response against the SARS-CoV-2 virus was undesirably affected by PRLT. The seroconversion rate and the absolute immune titers (in many cases >25,000 BAU/mL) are comparable to the normal population. This result implies the clinically important conclusion that neither an initial nor a booster vaccination against COVID-19 must be postponed if a PRLT is planned (and vice versa).

## Simple Summary

Cancer patients with weakened immune systems may struggle to fight off infections, and some treatments can make vaccines less effective. Researchers investigated whether a specific cancer treatment, radioligand therapy, affects the body’s ability to respond to COVID-19 vaccines. They examined records of 30 patients with advanced prostate cancer who had undergone this therapy and received COVID-19 vaccinations. Nearly all patients successfully developed immunity after their vaccinations, despite receiving radioligand therapy. Their immune response was comparable to the general population, suggesting that this cancer treatment does not negatively affect COVID-19 vaccine effectiveness. This finding is significant because it means that patients undergoing radioligand therapy do not need to delay their vaccinations. Protecting cancer patients from COVID-19 is crucial, and ensuring they can safely receive both treatment and vaccines without interference supports their overall health. The study provides reassurance to doctors and patients, emphasizing that life-saving cancer treatment and vaccinations can work together without reducing protection against the virus.

## 1. Introduction

One of the major challenges that all healthcare providers face during anti-cancer therapy is the side effects on the immune system, resulting in higher susceptibility to infections and secondary malignancies. Besides the risk of nosocomial infections with multi-resistant pathogens, infection with the severe acute respiratory syndrome coronavirus 2 (SARS-CoV-2) has become a major threat for immunocompromised cancer patients since the coronavirus disease 2019 (COVID-19) outbreak in 2020. The diminished immune state can result from the disease itself—as hematologic malignancies reduce the amount of functional white blood cells—but it can also be a side effect of the anti-cancer therapy that interacts with the function of immune cells or reduces their production in the bone marrow.

As different vaccines against SARS-CoV-2 became available at the end of 2020, physicians are often confronted with patients’ questions regarding the interference (in terms of efficacy and safety) of radioligand therapy (RLT) with the coronavirus disease 2019 (COVID-19) vaccination. Active immunization with the currently available vaccines as well as the immune response of matured COVID-19-specific immune cells require functional T- and B-lymphocytes, but at this moment, only little is known about the effects of RLT on the immune cells. The American Society of Clinical Oncology (ASCO) and the European Society of Medical Oncology (ESMO) released guidelines and strongly recommend vaccination of cancer patients, since cancer patients show a higher mortality compared to the general public if they become infected with COVID-19 [1,2]. For patients with malignancies, mortality rates reaching up to 36% have been reported in the literature [3]. Also, other expert groups recommended priority COVID-19 vaccination for cancer patients [4,5,6], except for certain contraindications, such as stem cell therapy and chimeric antigen receptor (CAR) T-cell therapy [7]. While the strong recommendation for vaccination is backed by studies across various treatment modalities, no data currently confirm the efficacy and safety of the COVID-19 vaccine in patients undergoing RLTs.

Since the vaccines became available, several studies have assessed the safety and efficacy profiles of different COVID-19 vaccines in diverse cancer patient populations receiving a range of treatment regimens, including single-agent or combination therapies with chemotherapeutics, biologics, hormonal therapy, immunotherapy, and radiotherapy [8,9,10,11]. To evaluate the efficacy of the COVID-19 vaccines in cancer patients, levels of neutralizing antibodies (representing the immediate humoral response) were measured. It must be noted that sufficient immunogenicity against SARS-CoV-2 is not only correlated with antibody titers but also depends on the immune response of memory B cells and T cells [12], and this factor was often not evaluated in the published studies. While the essentialness of immunological memory for long-term immunity is undoubted, the understanding of the interplay and the kinetics of the participating immune cells is still incomplete [13]. Nevertheless, antibody titers are clinically relevant biomarkers and a protective correlate for COVID-19 vaccines [14,15], but also, per definition, less pertinent to assess immunogenicity against new upcoming and distantly related mutants.

Several publications contemplated the effect of the diminished humoral response of COVID-19 vaccines in cancer patients [8,16] (including hematologic malignancies such as chronic lymphocytic leukemia (CLL) [17,18], C-cell non-Hodgkin lymphoma [19], and multiple myeloma [20]). In a study evaluating the humoral response after vaccination with the messenger ribonucleic acid (mRNA) COVID-19 vaccine BNT162b2 in patients with heterogenous malignancies, patients receiving chemotherapy were associated with significantly reduced neutralizing antibody titers after vaccination [21]. Only 81.3% of patients undergoing (unspecified) chemotherapy demonstrated serological response, in comparison to a 96.2% response rate in patients on other treatments, whereby vaccinated healthy control groups often achieved 98–100% seropositivity. It could also be shown that multiple-dose regimens (booster vaccinations) elicit seroconversion in cancer patients [22]. As the dynamics of SARS-CoV-2 neutralizing antibody responses and the longevity of immunity are greatly varying also in non-cancer patients [23], the assessment of antibody titers should become inevitable during active cancer therapy and aftercare. In conclusion, the current data support that vaccination of the vulnerable group of cancer patients is highly endorsed, whereby multiple vaccinations and serological surveillance are required to evaluate the level of protection against infection with COVID-19. Clinicians are advised to adhere to the updated COVID-19 vaccine immunization schedule published by authoritative organizations, such as the Centers for Disease Control and Prevention (CDC).

In this study, we aim to determine whether RLT affects the serological response to COVID-19 vaccination. While interactions between COVID-19 vaccination and various oncological treatments have been extensively studied over the past three years (vide supra), a significant gap remains in the literature regarding the potential impact of RLT on vaccine efficacy, including but not limited to COVID-19 vaccines. The COVID-19 outbreak and the concurrent vaccination campaign provided a unique opportunity for this investigation. Our patients were both naïve to the virus and motivated to undergo multiple vaccinations during their cancer treatment with RLT. Furthermore, due to this exceptional situation, our center routinely assessed both vaccination status and antibody titers in all patients. A study of this nature would not have been feasible with other vaccines, such as the influenza vaccine, due to the lower vaccination rates among our patient population and the higher costs associated with antibody testing. Additionally, routine COVID-19 testing was mandatory for every clinic visit, enabling both clinicians and patients to detect infections in real time—an approach not implemented for influenza or other infectious diseases. At the time, COVID-19 was also considered a significantly higher risk to cancer patients than influenza, further underscoring the relevance of our study.

To the best of our knowledge, no published data currently address the possible interference between RLT and immunogenicity against SARS-CoV-2. This retrospective single-arm study evaluates the effect of prostate-specific membrane antigen (PSMA)-targeted radioligand therapy (PRLT)—using either a single radionuclide (lutetium-177) or TANDEM therapy (lutetium-177 and actinium-225)—on immunogenicity in patients with metastatic castration-resistant prostate cancer (mCRPC).

## 2. Materials and Methods

### 2.1. Patient Selection and Characteristics

**Figure 1:**
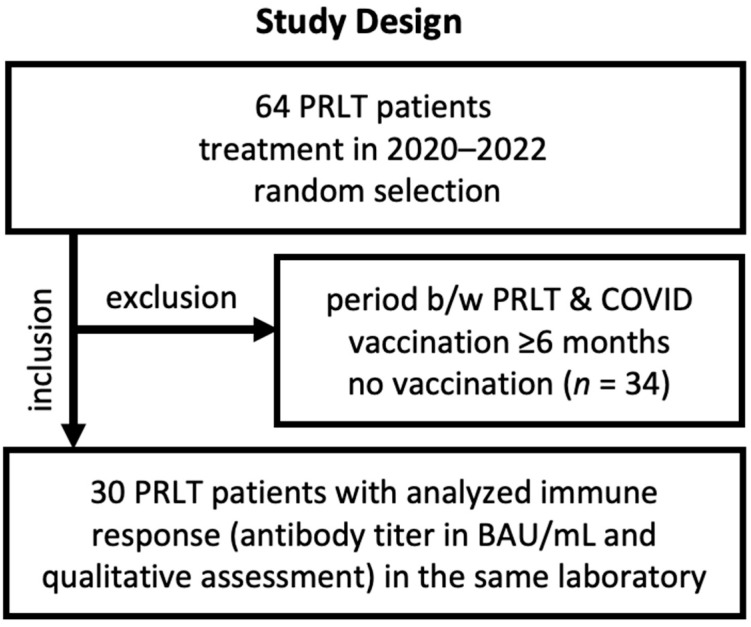
Study design with major inclusion and exclusion criteria.

For this retrospective, single-arm, and monocentric study, data on the immune status against COVID-19, patient characteristics including age, radioligand therapy regimen, vaccination history against COVID-19, and antibody level were retrospectively analyzed. Our database that included all mCRPC patients who were treated with PSMA-targeted radioligand therapy, 64 patient files (with PRLT cycles in 2020–2022) were randomly selected (Figure 1). Of 64 files, 34 files did not include any information on the serological status; information on vaccination status was missing or the patients did not receive a COVID-19 vaccination at all. In other cases, the period between RLT and vaccination was too long to expect any interference between both. The residual 30 patient files were analyzed. Table 1 presents the patient characteristics. As we assumed that any effect of RLT on the immune system will not last longer than 6 months (approx. 27 half-lives of lutetium-177 and 18 half-lives of actinium-225), we consider RLT cycles that were administered ≥6 months before COVID-19 vaccination not as a potential interfering treatment and do not expect any drop in immunogenicity.

In Table 1, the characteristics of the patient cohort (30 men with mCRPC) are summarized as follows: The mean patient age was 70.6 years, and therefore equal to the age of the patients in the VISION trial (phase 3 trial with lutetium-177-PSMA-617) [24]. Within the relevant period of June 2020 and July 2022 (note: as the first vaccination in Germany was administered in December 2020, we declare that any therapy cycle of PRLT earlier than June 2020 can be seen as minorly relevant), patients with known vaccination status received in average 2 cycles of PRLT (either lutetium-177-labelled PSMA-I&T, or a combination of lutetium-177- and actinium-225-labelled PSMA-I&T, ‘TANDEM therapy’). Within the cohort, 24 individuals received, on average, a cumulative dose of 16.1 ± 7.2 GBq (435.1 ± 194.6 mCi)/patient of lutetium-177-labelled PSMA ligand, whereby 6 patients additionally received a cumulative dose of 13.7 ± 6.6 MBq (0.37 ± 0.18 mCi)/patient of actinium-225-labelled PSMA ligand.

Until summer 2022, most of the patients had received a third (booster) vaccination and mostly mRNA vaccines were used for vaccination, but nevertheless, detailed information on the vaccine was missing in 27 cases of 81 administered doses (33%). Surprisingly, only 10% of the patients reported a past infection with COVID-19.

### 2.2. Evaluation of Serological Response

The serological analysis was performed at one laboratory; antibody levels are therefore standardized. A qualitative interpretation of the results was submitted to evaluate the serological response along (in nearly all cases) with the absolute SARS-CoV-2 antibody titer (in BAU/mL) (with ranges up to >25,000 BAU/mL). The different levels of the neutralizing antibodies were translated by the laboratory into terms such as excellent response, very good response, good response, still good response, very strong response, strong response, sufficient response, still sufficient response, indicating immune response, borderline response, insufficient response, and low titer. In Table 2, patients were stratified in seronegative (−), if their last evaluated antibody titer was described as ‘insufficient response’ or ‘low titer’ (the one patient with a borderline response is highlighted in Table 2).

## 3. Results

Table 2 summarizes the results of the serological immune response status against COVID-19 of vaccinated mCRPC patients that were treated—shortly before, during, or after vaccination—with either mono radionuclide (lutetium-177-radiolabelled) or TANDEM (actinium-225- and lutetium-177-radiolabelled) PRLT. In the analyzed cohort, 96.7% of the patients achieved seroconversion after receiving (on average) the third (booster) vaccination against SARS-CoV-2 and (on average) two cycles of RLT (with a lutetium-177 activity of 16.1 ± 7.2 GBq (435.1 ± 194.6 mCi)/patient and actinium-225 activity of 13.7 ± 6.6 MBq (0.37 ± 0.18 mCi)/patient (for TANDEM therapy)).

Over the course, serum analysis revealed an insufficient titer of neutralizing antibodies in four patients. Patient 3 received, six weeks before his first vaccination, the third cycle of lutetium-177-PSMA ligand therapy, followed by two vaccinations with the mRNA vaccine from BioNTech-Pfizer with a four-week gap. Approx. two months after the second vaccination, two cycles of TANDEM PSMA-directed ligand therapy were administered. However, half a year after the last vaccination and two months after the last RLT cycle, serum analysis revealed an insufficient antibody titer of 31 BAU/mL. The result is not surprising as the waning of titers is expectable after this time [23] in patients that did not receive a booster vaccination, therefore, the reason for insufficient immune response is unlikely attributable to the RLT. Notably, the last chemotherapy cycle with cabazitaxel was 18 months before his first immunization, and shortly thereafter, the patient was treated with the third cycle of TANDEM therapy (sixth of RLT in total). After nearly six months, the patient was boosted with BioNTech-Pfizer, and seroconversion was reached with a strong antibody titer (3524 BAU/mL). Similar dynamics were seen in patient 16: after initial vaccination, several serum analyses showed weaning of antibody levels over 6 months, and in this time, three courses of lutetium-177-PSMA RLT were administered. After receiving the booster immunization and one cycle of TANDEM therapy, a very high serological immune response was measured (13,389 BAU/mL), evidently showing that a strong immune response can be built up while receiving four cycles of RLT (30.1 GBq (813.5 mCi) cumulated activity of lutetium-177, 4.7 MBq (0.13 mCi) cumulated activity of actinium-225).

Patient 14 received his primary immunization with a mRNA vaccine from BioNTech-Pfizer (two doses within three weeks) and was boosted with the same vaccine after nine months. He received his first RLT half a year before his initial immunization and the second therapy cycle was administered, along with serum analysis, six months after the booster. Although guidelines for immunization were followed, the post-vaccination antibody titer was only 314 BAU/mL (54% neutralizing antibodies), indicating a low titer. Notably, the cut-off values were set from the laboratory; therefore, the low antibody level is assessed as seronegative even though specific antibodies could be detected and could also contribute to a protective immune response. The fourth patient with (transient) insufficient immune response (patient 19) received only one shot with the Johnson & Johnson (Janssen) vaccine, which was initially thought to establish a durable humoral response after one dose. Approx. six months before his vaccination, the patient was treated with the third cycle of RLT. After his vaccination, he received three further cycles over a one-year period, and during this time, serum analyses revealed an overall low antibody or insufficient antibody titer. Finally, seroconversion was achieved in patient 19 after a COVID-19 infection with relatively mild symptoms.

**Figure 2:**
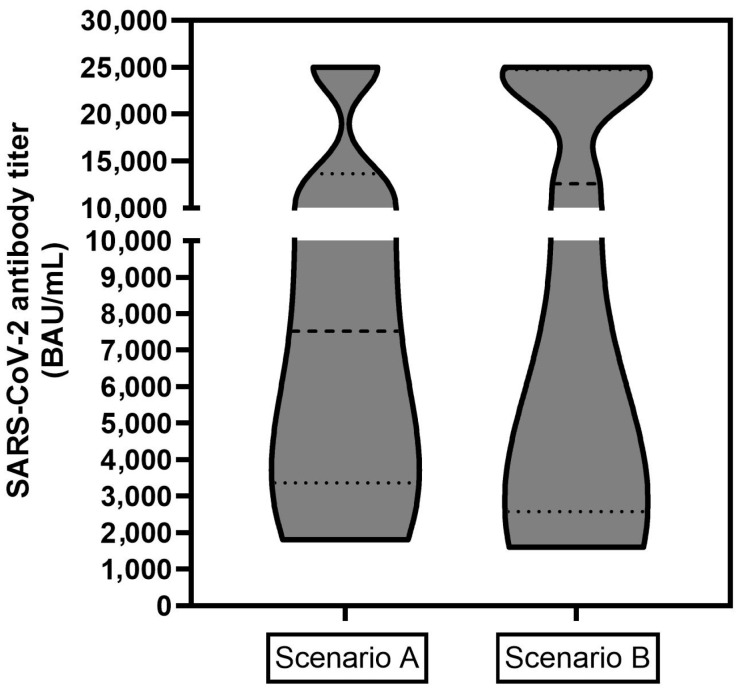
Absolute SARS-CoV2 antibody titers in BAU/mL for two clinical scenarios: Scenario A (left, *n* = 11), patient was vaccinated and subsequently treated with PRLT, the antibody response was evaluated a sufficient period after PRLT; Scenario B (right, *n* = 16): patient received vaccination(s) intermittently between PRLT treatment cycles and the antibody response was evaluated after a sufficient period after PRLT or vaccination. Titers of >25,000 BAU/mL represent the upper limit and were included as a value of 25,000 BAU/mL.

Also, the absolute antibody titers were quantitatively analyzed. Therefore, subjects were grouped into two different clinical scenarios. In scenario A (*n* = 11), the patients were vaccinated and subsequently treated with PRLT, and the antibody response was evaluated after a sufficient period subsequently to PRLT. In scenario B (*n* = 16, one subject with an unreasonable reported titer was removed), the patients received vaccination(s) intermittently between PRLT treatment cycles and the antibody response was evaluated after a sufficient period after PRLT or vaccination. As titers over 25,000 BAU/mL were not further diluted from the laboratory, this titer represents the upper limit in both plots represented in Figure 2. The third scenario (*n* = 2, vaccination after final PRLT, then determination of titer) led to insufficient/borderline (patient 10 and 14) titers and are not depicted in the graph (the case number is too low to draw any clinical conclusions).

## 4. Discussion

The data depicted in Table 1 shows clearly that 96.7% of the study population could build up a sufficient humoral immune response against COVID-19 while undergoing PRLT. In terms of antibody levels (Figure 2), no clear trend could be revealed and the absolute titers did not correlate with the sequence of PRLT and vaccination. As several subjects achieved, fortunately, titers with >25,000 BAU/mL, statistically accurate median or mean values (and error bars) could not be calculated which could reveal significant (or not significant) differences for both scenarios. Titers over 25,000 BAU/mL were achieved in patients with the best responses (*n* = 2 in scenario A, *n* = 4 in scenario B) while the lowest values were 1809 BAU/mL (scenario A) and 1600 BAU/mL (scenario B) and clinically rated by the laboratory as an at least sufficient immune response against the SARS-CoV19 virus.

A limitation of this study is the non-diverse population (elderly men with prostate cancer) and the sample size, which is restricted to patients who received PRLT between June 2020 and June 2022. This timeframe was chosen because, during this period, the strict testing protocols in German hospitals enabled the detection of subclinical COVID-19 infections. Additionally, antibody levels for each patient were analyzed as part of the routine laboratory panel. Furthermore, the number of administered vaccines and the prevalence of specific COVID-19 variants during this period were well-documented. In contrast, patients treated after this period would likely have received multiple COVID-19 vaccinations targeting various variants, making it difficult to draw conclusions about the development of immunogenicity during RLT.

Additionally, information on the vaccination status and antibody levels needed to be available. As the sample size was limited and no control group was established due to the retrospective nature, any in-depth correlations between vaccination and therapy regimen or administered activities could not be performed. However, as most individuals built up and maintained an immune response despite undergoing RLT, it can be hypothesized that the influence of RLT on the immunogenicity against SARS-CoV-2 is very minor and clinically neglectable. Notably, the crosstalk between RLT has been studied insufficiently so far, although the immunological effects of radiotherapy have already become a topic of increased interest [25].

Another limitation of our investigation is the narrow focus on serological response and not the investigation of events like (non-symptomatic) COVID-19 infections assessed by regular tests, but luckily, none of the patients reported any clinically severe COVID-19 infection. Additionally, seroconversion represents only an indicator of the humoral immune response and does not reflect the immunological contribution of T and B memory cells. While it is known that the waning of neutralizing antibodies shows high interindividual variability [23], the longevity of the cellular immune response is still a topic of investigation [13].

Due to the retrospective nature of the study, no data could be retrieved for a direct control group of prostate cancer patients undergoing earlier treatment lines (e.g., antihormonal therapy) or chemotherapy instead of PRLT, in combination with COVID-19 vaccination. Furthermore, our clinic does not typically treat or monitor patients receiving such alternative therapies. Patients on earlier treatment lines are also generally in better overall health, which could introduce a significant confounding factor.

In contrast, the strength of this study is that it represents the first investigation that examines the interference of RLT—using beta and also alpha emitters—with immunogenicity in a cohort with an increased risk for severe COVID-19 infections (mean age of 71 years in combination with metastatic cancer/terminal status). The finding might also be translatable to other vaccinations that are critical for terminal cancer patients (such as the recommended influenza shot) and to other RLTs such as somatostatin receptor therapy for the treatment of neuroendocrine tumors.

## 5. Conclusions

In conclusion, patients who undergo PSMA-targeted radioligand therapy do not have a decreased immunogenicity against SARS-CoV-2 if recommendations, such as the (first and/or second) booster vaccination with an mRNA vaccine [26,27,28], were followed. Therefore, the vaccination of these patients undergoing RLT with available SARS-CoV-2 vaccines seems to be safe and highly protective in over 96% of the patient population. Although the study did not include a control group of similar age, it is possible to compare the seroconversion quote in the general population (up to 95% after two vaccinations [29]). As mentioned earlier, other cancer treatments like chemotherapy significantly diminished the responder rate to only 81.3% [21] (studies on the efficacy of COVID-19 vaccinations in different cancer patient populations under treatment can be found in [8,9,10,11]). In this context, PSMA-directed RLT does not interfere with the development or maintenance of an immune response. These initial findings suggest that the critical COVID-19 vaccination does not need to be delayed if RLT is planned, and *vice versa* [30,31].

## Figures and Tables

**Table 1:** Patient characteristics.

	Patient Characteristics
Sex	Male = 30
Age ^1^	Mean: 70.6 years, Span: 56–86
Tumor	mCRPC
RLT cycles ^2^	Modal value: 2, Span: 1–4
Regimen	Patients receiving lutetium-177-PSMA-I&T: 24 Patients receiving lutetium-177-PSMA-I&T and/or TANDEM therapy: 6 Cumulative mean activity of lutetium-177: 16.1 ± 7.2 GBq (435.1 ± 194.6 mCi)/patient Cumulative mean activity of actinium-225: 13.7 ± 6.6 MBq (0.37 ± 0.18 mCi)/patient (TANDEM) Administered lutetium-177-PSMA cycles: 57 (1.9 per patient) Administered TANDEM cycles: 10 (1.7 per TANDEM patient)
Vaccinations	Modal value: 3, Span: 1–4
Vaccines (drug developer)	BioNTech-Pfizer:34 Moderna: 11 AstraZeneca-Oxford: 9 Johnson & Johnson (Janssen): 1 Unknown: 27 Confirmed COVID-19 infections: 4

^1^ at the last measurement of antibody levels. ^2^ any RLT cycle 6 months before the first vaccination. If the exact date of the first vaccination was unknown, any administered RLT after June 2020 is seen as relevant.

**Table 2:** Evaluation of serological response in the study population. AZ = AstraZeneca-Oxford, b: borderline response; BNT: BioNTech-Pfizer; C: past COVID-19 infection; JNJ: Johnson & Johnson (Janssen); MD: Moderna; Pat.: patient number; U: unknown; (+): seropositive, (−): seronegative.

		Radioligand Therapy (Cumulative Administered Activity)			COVID-19 Vaccination		
Pat.	Age ^1^	Cycles	Lutetium-177, GBq (mCi)	Actinium-225, MBq (mCi)	Doses	Vaccines (Drug Developer) or Infection	Seroresponse
1	85	2	15.1 (408.1)	-	3	BNT, BNT, BNT	+
2	69	3	22.5 (608.1)	-	1	C, BNT	+
3	53	4	20.1 (543.2)	23.7 (0.64)	3	BNT, BNT, BNT	+
4	65	2	16.1 (435.1)	-	2	BNT, BNT	+
5	69	2	15.6 (421.6)	12.0 (0.32)	3	U, BNT, BNT	+
6	73	2	18.0 (486.5)	16.7 (0.45)	3	U, U, BNT	+
7	85	2	13.4 (362.2)	-	3	U, U, BNT	+
8	71	2	15.5 (418.9)	-	3	U, U, BNT	+
9	73	2	18.4 (497.3)	-	2	MD, MD	+
10	65	1	8.7 (235.1)	-	3	AZ, AZ, BNT	+ (b)
11	78	2	17.5 (473.0)	-	3	C, U, U, BNT	+
12	68	2	14.5 (391.9)	-	3	U, AZ, MD	+
13	69	3	19.4 (524.3)	-	3	MD, MD, MD	+
14	63	1	6.3 (170.3)	-	3	BNT, BNT, BNT	-
15	77	1	12.8 (346.0)	-	2	C, MD, MD	+
16	70	4	30.1 (813.5)	4.7 (0.13)	3	U, BNT, BNT	+
17	66	2	17.4 (470.3)	-	3	U, AZ, BNT	+
18	68	2	16.0 (432.4)	-	3	AZ, AZ, MD	+
19	72	4	30.3 (818.9)	-	1	JNJ, C	+
20	86	3	23.9 (645.9)	-	3	BNT, BNT, BNT	+
21	62	3	15.2 (410.8)	-	3	U, U, BNT	+
22	74	2	15.5 (418.9)	-	2	BNT, BNT	+
23	66	1	8.9 (240.5)	-	3	U, U, BNT	+
24	75	3	7.5 (707.7)	-	3	U, U, U	+
25	74	1	7.5 (707.7)	-	4	U, U, AZ, MD	+
26	56	3	7.0 (189.2)	-	3	BNT, MD, MD	+
27	71	4	33.2 (897.3)	-	4	U, BNT, U, BNT	+
28	73	2	19.7 (532.4)	-	3	U, U, BNT	+
29	70	2	14.5 (391.9)	9.0 (0.24)	3	U, U, BNT	+
30	74	1	2.1 (56.8)	16.0 (0.43)	3	AZ, AZ, BNT	+

^1^ At last serum analysis.

## Data Availability

The data presented in this study are available on request from the corresponding author due to protecting patients’ personal information.

## Abbreviations

The following abbreviations are used in this manuscript:

ASCO	American Society of Clinical Oncology
CAR	chimeric antigen receptor
CLL	chronic lymphocytic leukemia
COVID-19	coronavirus disease 2019
CDC	Centers of Disease Control and Prevention
ESMO	European Society for Medical Oncology
mCRPC	metastatic castration resistant prostate cancer
mRNA	messenger ribonucleic acid
PRLT	PSMA-directed radioligand therapy
PSMA	prostate-specific membrane antigen
RLT	radioligand therapy
SARS-CoV-2	severe acute respiratory syndrome coronavirus 2
